# A technique for quantifying intracellular free sodium ion using a microplate reader in combination with sodium-binding benzofuran isophthalate and probenecid in cultured neonatal rat cardiomyocytes

**DOI:** 10.1186/1756-0500-6-556

**Published:** 2013-12-26

**Authors:** Daisuke Katoh, Kenichi Hongo, Keiichi Ito, Takuya Yoshino, Yosuke Kayama, Kimiaki Komukai, Makoto Kawai, Taro Date, Michihiro Yoshimura

**Affiliations:** 1 Division of Cardiology, Department of Internal Medicine, The Jikei University School of Medicine, Tokyo 105-8461, Japan

**Keywords:** Intracellular sodium, Cardiomyocyte, SBFI, Probenecid, Microplate reader

## Abstract

**Background:**

Intracellular sodium ([Na^+^]_i_) kinetics are involved in cardiac diseases including ischemia, heart failure, and hypertrophy. Because [Na^+^]_i_ plays a crucial role in modulating the electrical and contractile activity in the heart, quantifying [Na^+^]_i_ is of great interest. Using fluorescent microscopy with sodium-binding benzofuran isophthalate (SBFI) is the most commonly used method for measuring [Na^+^]_i_. However, one limitation associated with this technique is that the test cannot simultaneously evaluate the effects of several types or various concentrations of compounds on [Na^+^]_i_. Moreover, there are few reports on the long-term effects of compounds on [Na^+^]_i_ in cultured cells, although rapid changes in [Na^+^]_i_ during a period of seconds or several minutes have been widely discussed.

**Findings:**

We established a novel technique for quantifying [Na^+^]_i_ in cultured neonatal rat cardiomyocytes attached to a 96-well plate using a microplate reader in combination with SBFI and probenecid. We showed that probenecid is indispensable for the accurate measurement because it prevents dye leakage from the cells. We further confirmed the reliability of this system by quantifying the effects of ouabain, which is known to transiently alter [Na^+^]_i_. To illustrate the utility of the new method, we also examined the chronic effects of aldosterone on [Na^+^]_i_ in cultured cardiomyocytes.

**Conclusions:**

Our technique can rapidly measure [Na^+^]_i_ with accuracy and sensitivity comparable to the traditional microscopy based method. The results demonstrated that this 96-well plate based measurement has merits, especially for screening test of compounds regulating [Na^+^]_i_, and is useful to elucidate the mechanisms and consequences of altered [Na^+^]_i_ handling in cardiomyocytes.

## Findings

### Background

The sodium ion (Na^+^) is the main determinant of the body fluid distribution, and transsarcolemmal Na^+^ gradient is a key regulator of the various intracellular ions and metabolites. In the heart, the concentration of free intracellular Na^+^ ([Na^+^]_i_) has been shown to increase in the presence of cardiac diseases including ischemia, heart failure, and hypertrophy [1-5]. Because [Na^+^]_i_ is important in modulating the electrical and contractile activity, quantifying [Na^+^]_i_ is of great interest. Therefore, several techniques for measuring [Na^+^]_i_ have been established to clarify the mechanisms and consequences of altered [Na^+^]_i_ regulation, and the standard procedure currently used for measuring [Na^+^]_i_ in a single cell is a fluorescent microscopy-based method [6-10]. Sodium-binding benzofuran isophthalate (SBFI), the most widely used Na^+^-sensitive fluorescent indicator provides spatial and temporal resolution of [Na^+^]_i_ with sufficient selectivity in the presence of physiological concentrations of other ions [11]. The ratiometric measurement with SBFI permits us to cancel out variable dye concentrations in the cells and shares the same filter equipment used for the Ca^2+^ indicator, Fura-2. Although the use of microscopy and ratio imaging in combination with SBFI has some merits, including the fact that it requires a minimal number of cells, permits the discrimination against dye leaked out of the cells, and provides the ability to see indicator compartmentalization [6], this technique requires a fluorescence microscope equipment to switch between filters. Furthermore, it is difficult to test the effects of several types of compounds and/or compounds at several concentrations simultaneously. On the other hand, a method using a cell suspension loaded with fluorescent indicator in a cuvette recorded by a spectrophotometer has been reported, but it might not be adequate for living adherent cells. Moreover, when one measures [Na^+^]_i_ in cells using a closed culture space without a perfusion chamber system to wash out the dye leaked from the cells, this leaked dye reduces the accuracy of the measurements of [Na^+^]_i_[6].

Microplate readers with a 96-well format have been widely used in combination with various types of cell-based applications, including measuring the fluorescence intensity, because it employs a standardized rapid protocol for screening and examining multiple cell types and compounds, while requiring small amounts of materials. A method for measuring [Ca^2+^]_i_ in adherent cells attached to a 96-well microtiter plate using a microplate reader has been reported previously [12]. However, to our knowledge, no microplate reader-based method has previously been applied to measure [Na^+^]_i_ in cardiomyocytes in combination with SBFI. Moreover, there are few reports on the long-term effects of compounds on [Na^+^]_i_ in cultured cells, although rapid changes in [Na^+^]_i_ during a period of seconds or several minutes have been widely discussed. In comparison with adult cardiomyocytes, neonatal cells have the advantage of being easily cultured and having a longer viability. Therefore, we applied cultured neonatal rat ventricular cardiomyocytes (NRVM) in this system to examine the chronic effects of compounds on [Na^+^]_i_.

The aim of this study was to investigate a new method to measure [Na^+^]_i_ in NRVM attached to a 96-well microtiter plate using a microplate reader and to confirm the rational *in vivo* calibration method for SBFI in this system. We also investigated the effects of probenecid against dye leakage out of the cells. To confirm the reliability of this technique, the rapid effects of the Na^+^/K^+^ ATPase inhibitor, ouabain, on [Na^+^]_i_ were evaluated. We further examined the chronic effects of aldosterone on [Na^+^]_i_ in NRVM to illustrate the utility of the new method.

## Results and discussion

### Probenecid prevents the leakage of SBFI from cardiomyocytes

As SBFI-AM hydrolyzes, the 340/380 nm excitation ratio gradually increases [6]. In our preliminary experiment, the fluorescence intensity continued to gradually increase during the measurements, even after the 60-minute period that had been previously reported to allow for complete hydrolysis [6]. Di Virgilio, et al. reported that the consequences of dye (Fura-2) leakage were relevant for experiments in closed cuvettes, because secreted dye can account for a considerable percentage of the total fluorescence signal [13-15]. Because each well of the 96-well plate that we used in our experiment was also a closed space, the gradual increase of fluorescence intensity after recording for a 60-minute period, at which time the completion of hydrolysis was expected [6], was speculated to be the result of dye leakage. Probenecid, an organic anion transport blocker, has been reported to prevent Fura-2 leakage from cells, and this effect has also been reported for SBFI used to measure [Na^+^]_i_[16]. Cao, et al. reported the value of [Na^+^]_i_ in neocortical neurons, and demonstrated that several compounds induced changes in [Na^+^]_i_ using a microplate reader with a 96-well format [17]. However, they did not use probenecid in their experiments. They might have been able to successfully measure [Na^+^]_i_ in neocortical neurons without taking into account the dye leakage, because the significance of dye leakage from the cells depends on the cell line.

**Figure 1:**
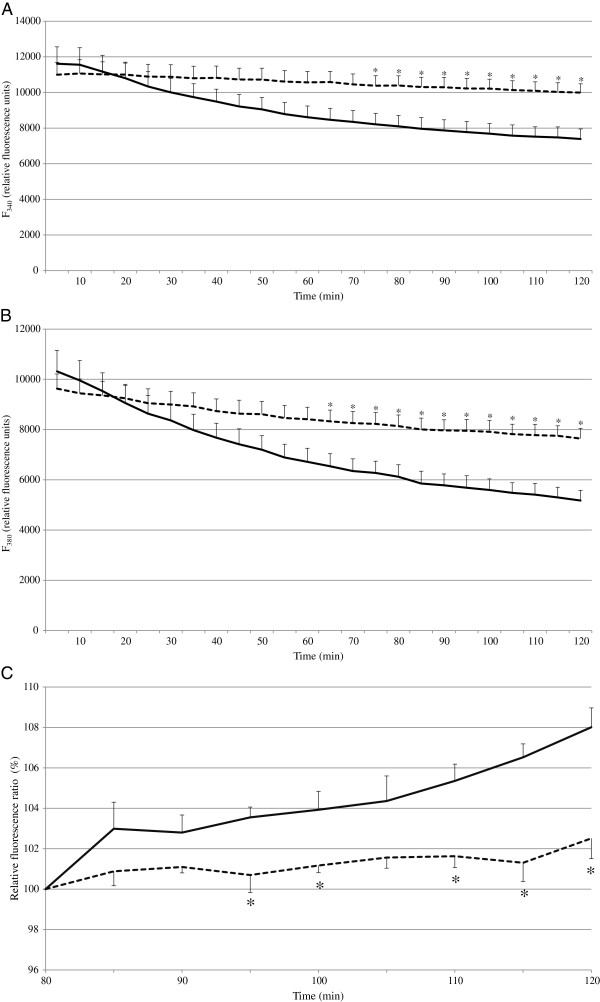
**Probenecid inhibits SBFI leakage from neonatal rat cardiomyocytes.** Adherent cardiomyocytes on a 96-well microplate were loaded with 5 μM SBFI-AM for 90 min at room temperature. Cells were washed twice and incubated in Tyrode solution in the presence or absence of 1 mM probenecid. The fluorescence intensity was measured every 5 min. **(A)** The time course of the background-subtracted SBFI fluorescence intensity at 340 nm (F_340_). **(B)** The time course of the background-subtracted SBFI fluorescence intensity at 380 nm (F_380_). **(C)** The relative SBFI fluorescence ratio (F_340_/F_380_), with the ratio at 80 min in each group considered to be 100%. *Solid lines*, without probenecid; *dashed lines*, with probenecid. *P < 0.05 vs cells treated without probenecid. The data are the means ± SE from four experiments.

To determine whether probenecid prevents dye leakage from cardiomyocytes in our 96-well microplate-based experiment, we compared the fluorescence ratio of SBFI in the cells incubated with the recording medium in the presence and absence of 1 mM probenecid. Because a stable SBFI fluorescence ratio was obtained after approximately 80 min of recording with 1 mM probenecid in the preliminary experiment, the relative fluorescence ratio compared to that at 80 min was estimated. Figures 1A and 1B clearly show the inhibitory effect of probenecid on the dye leakage from cardiomyocytes. A stable fluorescence ratio was obtained for at least 30 min after 80 min of recording in the presence of probenecid, while the ratio continued to increase in the wells without probenecid (solid line in Figure 1C. At 120 min recording, there was an estimated 8% increase in the SBFI ratio, indicating an approximately 8–10 mM increase in [Na^+^]_i_). This result indicates that probenecid is essential to prevent the overestimation of [Na^+^]_i_ caused by dye leakage. The concentrations of probenecid and time needed for treatment to inhibit dye leakage vary among different types of cells [13-15]. For our present method, probenecid effectively blocked SBFI efflux at a concentration of 1 mM, and was added only during the recording period after SBFI had been loaded into the cells. Therefore, in further experiments, we measured [Na^+^]_i_ in NRVM in Tyrode solution in the presence of 1 mM of probenecid. Several reports have suggested that probenecid can lead to unwanted effects in cells. In particular, probenecid has been reported to reduce the rise in [Ca^2+^]_i_ induced by depolarization of the plasma membrane or by a receptor-directed agonist, such as bradykinin [14]. Although this may not affect [Na^+^]_i_ itself, attention is needed for the function of cells when using this agent for a long time.

### Measurement of [Na^+^]_i_ and intracellular SBFI calibration in NRVM

**Figure 2:**
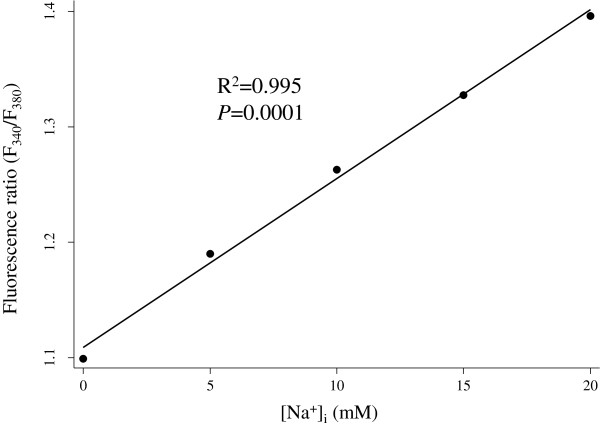
***In vivo *calibration of SBFI.** The *in vivo* calibration of SBFI was accomplished by exposing the myocytes to various extracellular [Na^+^]. Between 0 and 20 mM [Na^+^]_i_, the SBFI fluorescence ratio (F_340_/F_380_) showed a linear relationship with [Na^+^]_i_ (coefficient correlation (R^2^) = 0.995, *P* = 0.0001).

Figure 2 shows a typical *in vivo* calibration experiment for SBFI in NRVM. Between 0 and 20 mM [Na^+^]_i_, the SBFI fluorescence ratio (340/380 nm) showed a linear relationship with [Na^+^]_i_ (coefficient correlation (R^2^) of 0.995, with a slope of 0.14 ratio units per 10 mM change in [Na^+^]_i_). The value of [Na^+^]_i_ in NRVM calculated using this method was 7.5 ± 0.4 mM (n = 24), which is similar to the value in neonatal cardiomyocytes [18,19] and adult cells [9,20-22] measured by microscopy or a spectrophotometer, which ranged from 5 to 13 mM. These results suggest that our method has sensitivity comparable to the microscopy-based method. The value of [Na^+^]_i_ in myocytes depends on the ionic strength, pH, and the composition of the solutions used during the isolation of the myocytes [6,23,24]. In addition, the [Na^+^]_i_ levels in freshly prepared and cultured cells have been reported to be different for other cell lines [25]. Therefore, the protocol used needs to be carefully understood to ensure that an accurate comparison can be made of the absolute value of [Na^+^]_i_. Dye compartmentalization has been reported when SBFI is loaded at physiological temperature (37°C). However, this could be reduced by loading SBFI at room temperature [26,27]. In fact, the fraction of SBFI compartmentalized has been reported to range from 10 to 50% [7,8,10], and it is still uncertain even when it is recorded by microscopy, because the loaded dye concentration and loading time have varied among experiments. The disadvantages associated with population-averaged protocols using a plate reader and multiple cells, which thus meant that we could not directly detect indicator compartmentalization in the cells in each experiment are considered to be negligible, due to the fact that the changes in the fluorescence ratio are considered to mainly reflect the changes in the cytoplasmic [Na^+^] levels [6-8].

### Transient effects of ouabain on [Na^+^]_i_ in cardiomyocytes

**Figure 3:**
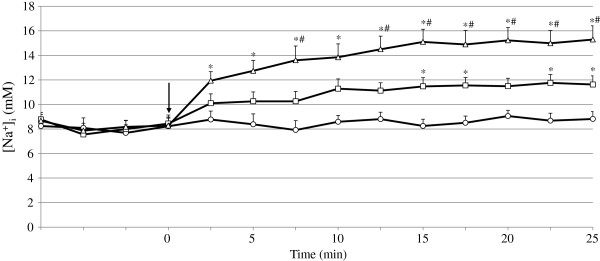
**Effects of ouabain on [Na**^**+**^**]**_**i **_**in cardiomyocytes.** The time-dependent changes in [Na^+^]_i_ during the treatment with vehicle (*open circle*), 100 μM ouabain (*open square*) and 200 μM ouabain (*open triangle*). The data are the means ± SE from 13 experiments. *P < 0.05 vs vehicle, #*P* < 0.05 vs 100 μM ouabain. The arrow indicates the application of compounds.

Ouabain is a specific Na^+^/K^+^ pump inhibitor that has been widely used for the treatment of patients with heart failure and atrial fibrillation. Ouabain is known to transiently alter [Na^+^]_i_ in cardiomyocytes. To confirm the reliability of our technique, we examined the effects of ouabain at concentrations of 100 μM and 200 μM on [Na^+^]_i_ in NRVM. During the application of ouabain, [Na^+^]_i_ increased significantly in a dose-dependent manner (Figure 3). The mean value of [Na^+^]_i_ at 20 min in the cells treated with vehicle, 100 μM and 200 μM ouabain was 9.0 ± 0.4 mM, 11.4 ± 0.6 mM and 15.2 ± 1.0 mM, respectively (n = 13). This result is comparable to the results reported using ouabain or another specific Na^+^/K^+^ pump inhibitor, strophanthidin, which were measured by fluorescent microscopy or a spectrophotometer [7,9,19], suggesting that our present technique detects the changes in [Na^+^]_i_ induced by agents with accuracy comparable to the traditional microscopy-based method.

### The long-term effects of aldosterone on [Na^+^]_i_ in cardiomyocytes

**Figure 4:**
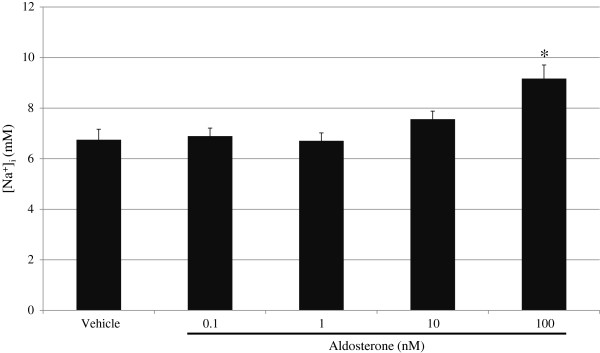
**The long-term effects of aldosterone on [Na**^**+**^**]**_**i **_**in cardiomyocytes.** Cardiomyocytes were treated with vehicle or aldosterone at a concentration of 0.1 nM to 100 nM for 24 h. The data are the means ± SE from 11 experiments. *P < 0.01 vs vehicle.

We and others have recently reported that aldosterone induces [Na^+^]_i_ elevation in cultured cardiomyocytes, and that this effect was rapid, non-genomic, and occurred in a mineralocorticoid receptor-independent fashion [28,29]. Although there was a previous report that aldosterone activated Na^+^/H^+^ exchange in cardiomyocytes [30], the long-term effect of aldosterone on the estimated value of [Na^+^]_i_ in cardiomyocytes is still unknown. To clarify this, we measured [Na^+^]_i_ in NRVM after treatment with vehicle or aldosterone at a concentration of 0.1 nM to 100 nM for 24 h using the new method. The mean value of [Na^+^]_i_ in cells treated with 100 nM aldosterone was significantly higher than that of cells treated with vehicle (9.1 ± 0.5 mM vs 6.7 ± 0.4 mM, n = 11, *P* < 0.01), although a lower concentration of aldosterone did not affect [Na^+^]_i_ (Figure 4). This result indicates that chronic aldosterone exposure alters [Na^+^]_i_ handling in cardiomyocytes, which might have (patho) physiological effects in the heart.

Most of the previous studies about [Na^+^]_i_ in the heart were focused on the rapid effects of agents. However, altered [Na^+^]_i_ handling under pathological conditions, including heart failure and cardiac hypertrophy, is a continuous phenomenon. In this context, using NRVM and investigating the change in [Na^+^]_i_ after long-term treatment with various compounds may be helpful for understanding the mechanisms and consequences of [Na^+^]_i_ handling in the heart.

## Conclusions

The results of this study indicate that using a microplate reader and a ratiometric measurement of SBFI used in combination with probenecid provides accurate values for [Na^+^]_i_ in NRVM attached to 96-well plates. This method has merits in that it allows for the changes in [Na^+^]_i_ in cultured cells treated with several types or concentration of agents to be measured simultaneously, and provides a more thorough investigation of the long-term effects of agents. In addition, the present method can be applied to measure [Na^+^]_i_ in other types of adherent cells with some modification of the concentration of probenecid and length of treatment.

## Methods

### Preparation of cardiomyocytes and cell culture

All animal procedures conformed to the National Institutes of Health Guide for the Care and Use of Laboratory Animals and were approved by the Animal Research Committee of Jikei University. NRVM were isolated from one- to three-day-old Sprague–Dawley rats according to the manufacturer’s protocol from Worthington Biochemical (Lakewood, NJ). Purified NRVM were plated at a density of 1*10^5^ cells/well in 96 well clear bottom plates in low-glucose (1000 mg/liter) DMEM (GIBCO) supplemented with 10% fetal bovine serum (GIBCO), 20 mM HEPES and antibiotics (100 U/ml penicillin G and 100 μg/ml streptomycin; Wako). The cells were allowed to attach at 37°C in a 5% CO_2_ atmosphere, and subconfluent myocyte monolayers were obtained after 48 h. Sixteen hours before treatments with the indicated agents, the medium was replaced with DMEM supplemented with charcoal-stripped FBS (GIBCO).

### Measurement of [Na^+^]_i_ in NRVM

NRVM were loaded with 5 μM SBFI-acetoxymethylester (AM) (Molecular Probes) dissolved in Tyrode solution (mM): 150 NaCl, 5.4 KCl, 1.2 MgCl_2_, 0.4 NaH_2_PO_4_, 10 HEPES, 5 glucose, and 1 CaCl_2_ (pH 7.4) for 90 min at room temperature in the presence of the non-ionic surfactant, Pluronic F-127 (0.05% w/v) [9,20]. After washing out the external dye twice with Tyrode solution, leaving a final volume of 200 μl in each well, the fluorescence intensity was measured by an Infinite 200 PRO microplate reader (TECAN) at room temperature. Dual excitation measurements at 340 nm and 380 nm were performed, and the emitted fluorescence was recorded at 510 ± 12.5 nm by the fluorescence bottom reading mode. The completion of hydrolysis was judged by attainment of a stable 340/380 nm ratio. In the experiments using ouabain, after attainment of a stable fluorescence ratio, we replaced 50 μl of medium in each well with 50 μl of a ouabain-containing solution. The microplate reader can take measurements in each well of a plate within 90 seconds, and the fluorescence intensity was automatically recorded every 2.5-5 minutes. In each microplate, NRVM of the same preparation in 10 wells were prepared with Tyrode solution in the absence of SBFI to measure the background signals of NRVM and microplates. Mean fluorescence signals from the 10 SBFI-unloaded wells at 340 nm and 380 nm were subtracted from the individual signals of SBFI-loaded wells at each wavelength. All of the experimental conditions, including *in vivo* calibrations, were performed in sextuplicate.

### *In vivo* calibration of SBFI

The *in vivo* calibration of SBFI was accomplished, similar to the previous reports, by exposing the cardiomyocytes to various concentrations of extracellular [Na^+^] (0–20 mM) in the presence of 1 mg/l gramicidin D, 100 μM strophanthidin, 2 mM EGTA, and the pH was adjusted to 7.1 with Tris base [9,20]. Myocytes had been treated with gramicidin D to allow the free movement of Na^+^, K^+^, and H^+^, strophanthidin to inhibit the Na^+^/K^+^ pump, and EGTA to increase the permeability of the cell membrane to Na^+^[6,8,9]. Using these agents, a stable equilibrium between the intracellular and the extracellular [Na^+^] was achieved. A linear fit of the calibration plots between 0 and 20 mM [Na^+^]_i_ was used to convert SBFI fluorescence ratios (340/380 nm) to values of [Na^+^]_i_. The calibration solutions were prepared by mixing two solutions of equal ionic strength. One solution contained 145 mM Na^+^ (30 mM NaCl, 115 mM sodium gluconate) and no K^+^, while the other one had 145 mM K^+^ (30 mM KCl, 115 mM potassium gluconate) and no Na^+^. Under these calibration conditions, the effect of K^+^ on SBFI is negligible in physiological [Na^+^]_i_ between 0 and 20 mM, although SBFI is known to be sensitive to K^+^[9]. A calibration was performed at the end of each experiment.

### Statistical analyses

The data are expressed as the means ± standard error for the indicated number of experiments. The statistical analyses were performed using Student’s t test and one way ANOVA, followed by Scheffe's test. Values of *P* < 0.05 were considered to be significant.

## Competing interests

The authors declare no competing interests.

## Authors’ contributions

DK, TY, KI performed experiments; DK, KH, KK, MK, TD and MY designed experiments; DK, KH, YK and MY wrote manuscript. All authors read and approved the final manuscript.
